# Hypertensive acute heart failure: a critical perspective on definition, epidemiology, pathophysiology, and prognosis—a narrative review: a joint session with the Romanian Society of Cardiology (part II)

**DOI:** 10.1007/s10741-025-10551-w

**Published:** 2025-09-02

**Authors:** Oliviana Geavlete, Sean P. Collins, Alexandre Mebazaa, Linda Ye, Alberto Palazzuoli, Laura Antohi, Jan Biegus, Matteo Pagnesi, Petar Seferovic, Razvan I. Radu, Avishay Grupper, Oscar Miro, Beth Davison, Magdy Abdelhamid, Marija Polovina, Mitja Lainscak, Marianna Adamo, Gad Cotter, Gianluigi Savarese, Mehmet Birhan Yilmaz, Maurizio Volterani, Giuseppe M. C. Rosano, Javed Butler, Andrew P. Ambrosy, Ovidiu Chioncel

**Affiliations:** 1 Emergency Institute for Cardiovascular Diseases ‘Prof. C.C. Iliescu’, 258 Fundeni Street, 2 District, 022328 Bucharest, Romania; 2 University of Medicine Carol Davila, Bucharest, Romania; 3 Department of Emergency Medicine, Vanderbilt University Medical Center and Veterans Affairs Tennessee Valley Healthcare System, Geriatric Research, Education and Clinical Center (GRECC), Nashville, TN USA; 4 Department of Anesthesiology and Critical Care and Burn Unit, Saint-Louis and Lariboisière Hospitals, FHU PROMICE, DMU Parabol, APHP Nord, Université Paris Cité, MASCOT, Paris, France; 5 Department of Medicine, Kaiser Permanente San Francisco Medical Center, San Francisco, CA USA; 6 Cardiovascular Diseases Unit CardioThoracic and Vascular Department, Le scotte Hospital Siena, University of Siena, Siena, Italy; 7 Department of Cardiology, Clinical Department of Intensive Cardiac Care, Wroclaw Medical University, Faculty of Medicine, Institute of Heart Diseases, Wroclaw, Poland; 8 Institute of Cardiology, ASST Spedali Civili, Department of Medical and Surgical Specialties, Radiological Sciences, and Public Health, University of Brescia, Brescia, Italy; 9 Serbian Academy of Sciences and Arts; Medical Faculty, University of Belgrade, Belgrade, Serbia; 10 Department of Cardiology, University Clinical Centre of Serbia, Belgrade, Serbia; 11 Faculty of Medical and Health Sciences, Tel-Aviv University, Department of Cardiovascular Medicine, Shamir Medical Center, Be’er Ya’akov, Israel; 12 Emergency Department, Hospital Clínic, IDIBAPS, University of Barcelona, Barcelona, Catalonia Spain; 13 Momentum Research Inc, Durham, NC USA; 14 INSERM UMR‑S 942 (MASCOT), Université Paris Cité, Paris, France; 15 Heart Initiative, Durham, NC USA; 16 Faculty of Medicine, Kasr Al Ainy, Department of Cardiovascular Medicine, Cairo University, Giza, Egypt; 17 Faculty of Medicine, University of Belgrade, Belgrade, Serbia; 18 Division of Cardiology, General Hospital Murska Sobota, Rakican, Slovenia; 19 Faculty of Medicine, University of Ljubljana, Ljubljana, Slovenia; 20 Department of Clinical Science and Education, Södersjukhuset, Karolinska Institutet, Stockholm, Sweden; 21 Faculty of Medicine, Department of Cardiology, Dokuz Eylul University, Izmir, Turkey; 22 IRCCS San Raffaele Roma, Cardiopulmonary Department, 00166 Rome, Italy; 23 San Raffaele Open University of Rome, Rome, Italy; 24 IRCCS San Raffaele Roma, Rome, Italy; 25 Baylor Scott and White Research Institute, Dallas, TX and University of Mississippi, Jackson, MS USA; 26 Department of Cardiology, Kaiser Permanente San Francisco Medical Center, San Francisco, CA USA; 27 Division of Research, Kaiser Permanente Northern California, 4480 Hacienda Drive, Pleasanton, CA 94588 USA

**Keywords:** Hypertensive acute heart failure, Systolic blood pressure, Definition, Management

## Abstract

Hypertensive acute heart failure (HT-AHF) has historically been recognized as a distinct clinical phenotype of AHF, characterized by acute pulmonary congestion in the context of elevated systolic blood pressure (SBP), typically > 140 mmHg. However, emerging evidence has begun to challenge the diagnostic accuracy, clinical utility, and relevance of this category. A main criticism of HT-AHF is its considerable overlap with other AHF clinical profiles, including acute decompensated heart failure (ADHF) and acute pulmonary oedema (APO). Clinical features such as dyspnea and pulmonary congestion are not unique to HT-AHF. Additionally, some HT-AHF patients concurrently fulfill diagnostic criteria for the ADHF phenotype, including a history of HF or signs of volume overload, leading to ambiguity in diagnosis. HT-AHF is associated with very low in-hospital mortality (0–2%) compared to other AHF phenotypes. Notably, there is no robust evidence linking high SBP to poor short- or long-term outcomes, nor are there randomized clinical trials validating distinct management strategies for HT-AHF. Often associated with the management of HT-AHF, vasodilators have shown limited benefit across trials, contributing to a downgrade in guideline recommendations. The relatively favorable short-term prognosis and the lack of a standardized, evidence-based treatment approach weaken the rationale for classifying HT-AHF as a standalone AHF category. Given the heterogeneity of clinical presentations, overlap with other AHF phenotypes, and lack of prognostic distinction or targeted therapy, the term “AHF with high SBP at presentation” offers a more flexible and clinically meaningful descriptor, encouraging a more nuanced approach to treatment.

## Introduction

Acute heart failure (AHF) represents a life-threatening clinical condition characterized by a rapid onset or gradual worsening of a broad spectrum of signs and symptoms that reflect volume and pressure overload and/or low cardiac output (CO) [1].

Although numerous classification schemes for AHF exist in the literature, based on various criteria, a clinical phenotype-based classification is crucial for understanding the pathophysiology of and making medical decisions during AHF presentation and hospitalization [2]. In terms of classification and definition of AHF clinical phenotypes, very few registries [2–5] described specifically the hypertensive acute heart failure (HT-AHF) phenotype. Notably, there are no prior clinical trials that specifically include the HT-AHF population, nor any current randomized clinical trials that are specifically enrolling HT-AHF patients.

Among the clinical AHF phenotypes, hypertensive acute heart failure (HT-AHF) appears to be the most peculiarly characterized phenotype in terms of epidemiology, diagnostic criteria, pathophysiology, management, and even prognostic outcomes compared to the other subtypes [2]. Despite the 2012 and 2016 European Guidelines for the Diagnosis and Treatment of HF recognizing HT-AHF as a distinct clinical phenotype, the 2021 HF Guidelines classification did not include HT-AHF due to its significant overlap with other clinical profiles, such as acute pulmonary oedema (APO) and acute decompensated heart failure (ADHF), and the very low in-hospital mortality associated with this particular profile [1, 2].

Conventionally, HT-AHF refers to a rapid onset of dyspnea in patients with systolic blood pressure (SBP) > 140 mmHg, nearly all of whom have poorly controlled chronic hypertension (HTN) [2–5]. Although an increased prevalence of HTN in AHF settings is widely described in the literature, not all patients with AHF and high BP at presentation have HT-AHF, and not all HT-AHF patients have high SBP as a precipitant for AHF. This raises several limitations regarding the accuracy of the definition and may require the inclusion of multiple additional criteria beyond SBP, such as clinical presentation (“de novo” vs. worsening HF), etiology, and type of precipitant. In addition, multiple factors must be integrated into the overall clinical picture, including left ventricular (LV) systolic and diastolic function, baseline volume status, the magnitude of BP surge, the impact on target organs, and the degree of respiratory distress.

This paper focuses on the epidemiology, diagnosis, pathophysiology, and management of patients with HT-AHF. In addition, the authors offer a critical perspective on the definition of HT-AHF, drawing on the recent ESC-HF Guidelines [1]. In the present manuscript, the study’s inclusion criteria for the definition and classification of the HT-AHF phenotype were national or global AHF registries and studies published after 2005, enrolling a cohort size of over 1000 patients and providing outcome data availability.

## Classifying patients and definitional challenges

Data from US registries enrolling AHF patients reported a high prevalence of HTN, stating that 50% and 25% of AHF patients have SBP at presentation > 140 mmHg and > 160 mmHg, respectively [6, 7].

The association between AHF and HTN was used to classify AHF patients for the first time in the Euro-Heart Failure Survey II (EHFS II) [5]. The 2008 ESC HF Guidelines defined HT-AHF as “signs and symptoms of HF accompanied by high BP and usually relatively preserved LV function.” The patients are often euvolemic or mildly hypervolemic but frequently present with signs of pulmonary congestion without signs of systemic congestion [8]. Later, several large registries [2–4], consensus documents [9, 10], and reviews [11–13] considered HT-AHF a distinct phenotype when classifying patients with AHF.

While 35–50% of all AHF patients may exhibit an SBP > 140 mmHg, not all cases are categorized as HT-AHF [14]. As previously mentioned, HT-AHF is defined as the rapid onset of pulmonary congestion in the setting of an SBP > 140 mmHg. The sudden onset of symptoms is what distinguishes this phenotype from other forms of AHF [14]. Alternatively, a more restrictive characterization of HT-AHF is the presence of severely elevated SBP (160–180 mmHg) and no other cause of AHF except HTN [10, 15].

A hypothetical SBP threshold capable of producing AHF decompensation is difficult to define and exhibits large inter-individual variability. In patients with limited cardiac reserves, only a small increase in SBP may lead to significant worsening of the signs and symptoms of HF. For these patients, the acuity of change or the rate of increase of SBP may be more important. Opposite, AHF decompensation in patients with preserved cardiac reserves requires substantially high SBP values.

**Fig. 1:**
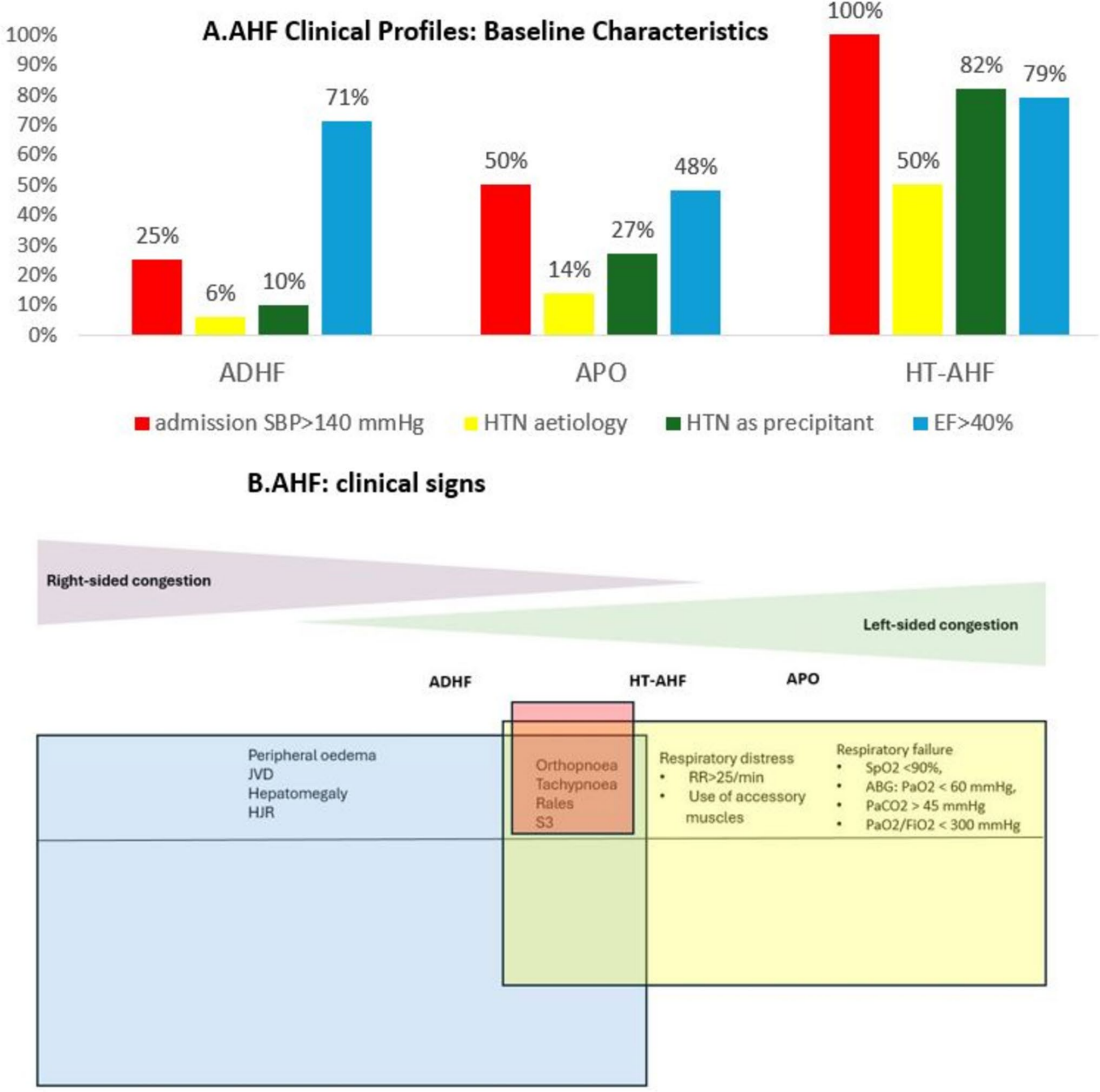
Overlapping among ADHF, HT-AHF, and APO. **A**. Investigator-rated classification of AHF *(ref 2)*. Components of the definition of HT-AHF (SBP at admission>140mmHg, HTN as precipitant, HTN aetiology, EF>40%) are seen in various proportions in other clinical profiles, suggesting low accuracy of the investigator-rated classification of AHF. **B**. Clinical signs of pulmonary and systemic congestion can overlap between AHF clinical profiles. Although HT-AHF typically manifests with pulmonary congestion, clinical signs of systemic congestion may also be present in HT-AHF. Both APO and HT-AHF present with signs of pulmonary congestion; however, the distinction between them is primarily related to respiratory distress and respiratory failure, which are diagnostic features of APO. Abbreviations: *ADHF* acute decompensated heart failure; *ABG* arterial blood gas; *APO *acute pulmonary oedema; *EF *ejection fraction; *HT-AHF *hypertensive acute heart failure; *JVD *jugular vein distension; *HJR *hepatojugular reflux; *RR* respiratory rate; *SBP *systolic blood pressure; *S3* sound 3

However, AHF classification in the 2021 ESC-HF Guidelines included only four clinical phenotypes: ADHF, APO, cardiogenic shock (CS), and isolated RV failure, without any mention of HT-AHF. The exclusion of HT-AHF from the 2021-ESC-HF Guidelines as a distinct clinical entity primarily stems from its significant clinical overlap with other clearly defined AHF phenotypes, such as ADHF and APO (Fig. 1). While the pathophysiological mechanisms differ between HT-AHF and other AHF phenotypes (congestion in APO and ADHF occurs at a primarily lung or systemic level, while congestion in HT-AHF occurs at a vascular level where LV-aortic uncoupling leads to insufficient ability to compensate for increases in afterload and preload, thereby causing elevated LV filling pressure), the resulting clinical profile is very similar [12]. Therefore, the HT-AHF profile often presents a clinical dilemma, as its symptomatology and management strategies substantially overlap with other AHF phenotypes despite differing pathophysiological mechanisms. This makes it difficult to identify as a unique, distinct category without redundancy. Also, irrespective of clinical phenotype, diagnostic tools for AHF include clinical examination, natriuretic peptides, lung ultrasound, chest X-ray, and echocardiography. In selected cases, right heart catheterization provides direct hemodynamic assessment and may identify elevated filling pressures or preserved output when diagnostic uncertainties persist, particularly in patients with HFpEF [16].

Additionally, the mortality rates associated with HT-AHF are generally lower when compared to other AHF-recognized profiles. This lower risk profile further influences the guideline’s approach to prioritizing classifications that more effectively encapsulate higher-risk populations. By focusing on classifications that reflect more severe clinical profiles, the guidelines aim to guide treatment protocols that can be applied urgently and address the most relevant clinical presentations.

In patients with a history of HF, the diagnosis of HT-AHF is based on the classic symptoms and signs of HF in conjunction with long-standing HTN and evidence of LV hypertrophy (LVH), where increased arterial resistance leads to fluid redistribution into the interstitial tissue [17]—a clinical profile that closely parallels heart failure with preserved ejection fraction (HFpEF) [18], which in turn is highly prevalent among patients with HT-AHF.

## Epidemiology

Arterial hypertension is the most common modifiable risk factor for HF, and its duration has a significant impact on the risk of developing HF [19]. The Framingham Heart Study showed a direct correlation between the cumulative incidence of HF and the severity of HTN in patients between 60 and 69 years after a follow-up period of over 14 years [20]. The lifetime risk for HF in HTN patients ranges from 20 to 45%, depending on sex and race, but if other conventional cardiovascular risk factors are associated with the HTN, such as diabetes or obesity, the risk of progression to HF increases up to 86% in 30 years [21, 22]. Long-term treatment of HTN reduces the risk of HF by ∼50% and is associated with lower HF mortality [19]. In addition, uncontrolled HTN is a well-recognized precipitating factor for AHF.

In European registries considering this phenotype, HT-AHF has a reported prevalence of 4–11% [2, 4, 5, 23] (Table 1). Notably, these estimates are based on investigator-assessed classifications of AHF. Furthermore, while the proportion of patients initially classified as having HT-AHF is generally higher in older registries (11.4% in EHFS II), there is also considerable geographic variation in the classifications [5]. These geographical differences are explained by variations in interpreting the definitions and cultural perceptions of severity, with differing thresholds for hospital admissions.

**Table 1:** Baseline characteristics, in-hospital treatment, and mortality in AHF registries considering HT-AHF as a distinct phenotype

	**RO-AHFS**^**3**^	**EHFS II**^**4**^	**ESC-HF LT**^**2**^	**AHEAD**^**5**^
Country/year/total number of patients with AHF	**Romania** **2011/3224**	**Europe** **2006/3580**	**Europe** **2017/6629**	**Czech Republic 2011/4153**
HT-AHF (% out of total)	**5**	**11.4**	**4.84**	**4.31**
Demographics				
Mean age (*y*)	71.5 ± 9.8	69.8	NA	74.8
Men	39	60.4	50	34.6
Medical history				
HTN (%)	95.6	94.6	81.5	94.3
Dyslipidemia (%)	47.8	NA	NA	NA
Diabetes mellitus (%)	34	34.5	35.6	43.1
Smoking (%)	20.1	NA	NA	NA
Previous MI (%)	8.7	NA	37.8	26.4
Vitals				
HR (beats/min)	98.8 ± 28.5	95	87.5	93
SBP (mm/Hg)	143.3 ± 39	170	170	198
Clinical signs				
Pulmonary rales (%)	NA	NA	66.7	NA
Peripheral oedema (%)	NA	NA	40.9	NA
JVP > 6 (%)	NA	NA	20.6	NA
Pulmonary congestion (%)	72	NA	66.7	NA
Peripheral congestion (%)	40	NA	38.2	NA
IV therapies/procedural interventions				
Diuretics	69.2	68.6 + 5.5	73.3	47.7
Nitrates	38.4 (33)	39.7	36.9	16.9
Inotropes/vasopressors (total %)	0	5.1	1.5	6.8
IABP	0	0.5	0	NA
PCI/CABG	1.9	7/0.5	5	NA
ICD/PM	0	2.9/1.7	5.3	NA
Hospital course				
Median LOS (d)	NA	8	NA	NA
In-hospital ACM	NA	NA	NA	NA
In-hospital mortality	0	1.4	1.8	2.2

Unless otherwise indicated, all values are reported as a percentage (%)

*EHFS II* Euro Heart Failure Survey II, *HTN* hypertension, *HTN-HF* hypertensive heart failure, *RO-AHFS* the Romanian Acute Heart Failure Syndromes, *ESC-HF-LT* European Society of Cardiology Heart Failure Long-Term Registry, *ADHF* acute decompensated heart failure, *APO* acute pulmonary oedema, *CS* cardiogenic shock, *RV-HF* right ventricle heart failure, *y* years, *HR* heart rate, *SBP* systolic blood pressure, *NA* not available, *PCI* percutaneous coronary intervention, *CABG* coronary artery by-pass graft, *ICD* implantable cardioverter defibrillator, *PM* pacemaker, *LOS* length of in-hospital stay

PCI ± CABG

Most HT-AHF patients had a history of HTN, which was considered the primary etiology of AHF. HTN was identified as the underlying cause in 94.6% of patients with HT-AHF in the EHFS II survey [5], in all patients in the RO-AHFS registry [3], and 50.3% of those included in the ESC-HF-LT Registry [2]. Uncontrolled HTN served as an AHF precipitating factor in 82% of cases in the ESC HF-LT Registry [2] (Table 1).

Notably, chronic kidney disease (CKD) is also common in this population and can act as a precipitating factor in HT-AHF. Renal dysfunction in CKD disrupts cardiovascular homeostasis through dysregulation of neurohormonal activity and vascular and myocardial remodelling, leading to HTN and, ultimately, increasing the risk of decompensation [24]. In a post hoc analysis of RELAX-AHF-2 investigating the association between various multimorbidity subtypes and clinical outcomes of AHF patients, the authors found that CKD was the second most prevalent comorbidity in HF patients, after HTN [25]. Additionally, the association between diabetes and CKD was very common in AHF and associated with all-cause mortality and rehospitalization for HF [26].

Several registries have reported signs of fluid overload in HT-AHF, including dyspnea, rales, and other evidence of pulmonary congestion. In the ESC-HF-LT Registry, of the patients classified as HT-AHF, 21% had jugular venous pressure (JVP) > 6, 41% had peripheral oedema, and 18% had hepatomegaly [2]. In a sub-analysis of the RO-AHFS registry [23], at admission, 40% of HT-AHF patients had signs of systemic congestion, and 13% presented with significant weight gain (Table 1). De novo AHF was the predominant presentation in HT-AHF patients (73.3% in the ESC HF-LT [2] Registry, 82.8% in EHFS II [5], and 74.3% in the AHEAD Registry [4]).

More than half of the patients enrolled in registries had preserved LV ejection fraction (LVEF > 45%): 91.8% in RO-AHFS [23], 53.4% in ESC-HF-LT [2], and 50.7% in EHFS II [5] (Table 1). LVH was present in 42.1% of patients with HT-AHF in the RO-AHFS Registry [23].

In-hospital mortality varied between 0 and 2%, the lowest among the other clinical profiles of AHF. However, 1-year mortality and HF hospitalization were 12% and 14%, respectively [2].

The data from RCTs are very scarce, as no RCTs specifically enrolled patients with HT-AHF.

Based on these results and the lack of benefit of vasodilator use in RCTs, nitrates were downgraded from Class IA to Class IIB recommendation in the 2021 guidelines for HF [1]. They are indicated as an initial treatment in patients with AHF and SBP > 110 mmHg in the setting of APO, aiming to reduce LV afterload [1].

There are no RCTs to demonstrate an association between high SBP and short-term and long-term outcomes. In a large cohort of 56,942 older patients hospitalized for HF, the investigators found an independent and continuous relationship between higher first SBP measurement and lower short- and long-term mortality [27]. The association was present across the entire spectrum of SBP, even at the highest levels, and was consistent among patient groups. In a pooled analysis of four AHF trials with serelaxin, a moderately elevated SBP (136–145 mmHg) was significantly associated with lower 180-day mortality as compared to normal SBP (125–135 mmHg) [28].

## Pathophysiologic considerations of HTN and its implications in AHF

**Fig. 2:**
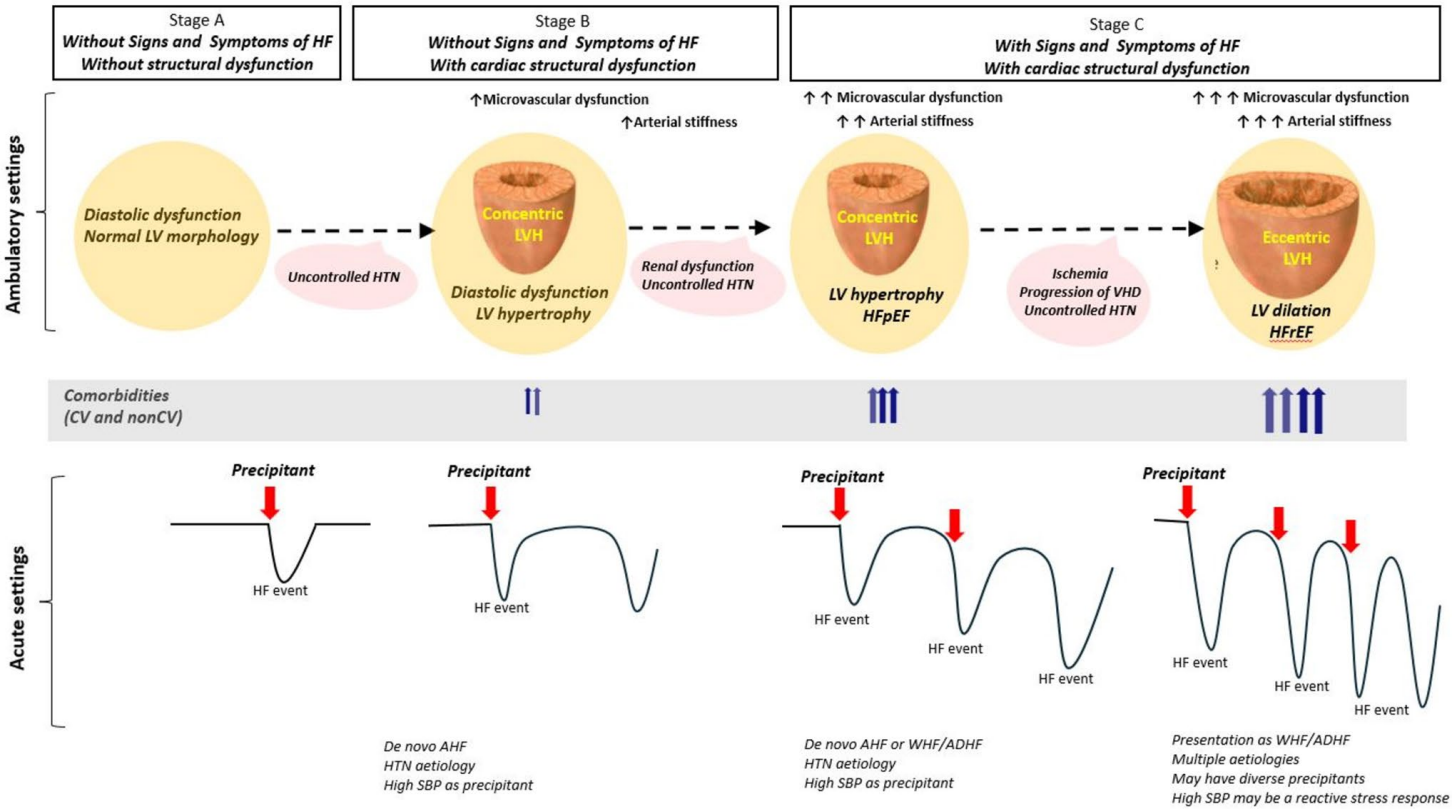
Chronological structural and functional changes in longstanding HTN and association with HF stages. AHF with high SBP at admission may be present in all stages. In stages A and B, HTN determines cardiac functional abnormalities, but with a normal structural LV. Any precipitant, particularly high SBP, can lead to an HF event. Further on, uncontrolled HTN will lead to cardiac structural changes, microvascular dysfunction, and arterial stiffness. In these scenarios, HTN can be both the cause and the precipitant for “de novo” AHF. In stage C, the worsened course of symptomatic HF is the consequence of the progression from LVH to LV dilatation and may present as HFpEF or HFrEF. Various precipitants (not only high SBP) may destabilize the clinical condition and lead to worsening HF. Furthermore, as HF progresses, several cardiac (e.g. ischemia, atrial fibrillation, valvular heart diseases) and non-cardiac comorbidities (e.g. diabetes, anemia) may impact clinical worsening. Although HTN was the primary etiology in these stages, many other etiologies contribute to the pathophysiology of HF. Abbreviations: *AHF* acute heart failure; *HTN* hypertension; *LV *left ventricle; *LVH* left ventricular hypertrophy; *HFpEF* heart failure with preserved ejection fraction; *HFrEF* heart failure with reduced ejection fraction; *SBP* systolic blood pressure

Hypertensive pathophysiology secondary to longstanding HTN represents an acceptable model for the interplay between the cardiovascular substrate and precipitants in AHF [29] (Fig. 2). Maladaptive changes in the neurohormonal system, myocardium, and vasculature in response to chronic HTN create a complex system sensitive to pressure, volume, and sympathetic tone changes [30, 31]. Sustained afterload increase from chronic HTN leads to a decrease in LV compliance, thereby causing diastolic dysfunction and subsequent structural remodelling with concentric LVH and, in later stages, LV dilation [32].

From a pathophysiological standpoint, HT-AHF’s primary insult is traditionally considered an acute increase in afterload. This is often amplified by a rapid fluid shift from the splanchnic veins into the pulmonary circulation. The splanchnic veins are compliant and have predominantly alpha-1 adrenergic receptors. The sympathetic stimulation of splanchnic veins results in pronounced vasoconstriction and rapid mobilization of up to 800 mL of blood into the systemic circulation [33, 34]. While this phenomenon of fluid redistribution might be encountered in patients with “vascular failure” and ventriculo-arterial stiffening leading to high-pressure-load dependence, progressive volume overload due to persistent neurohormonal activation is thought to play the central role in patients with severely impaired LV systolic performance or “cardiac failure” [35]. In these cases, only mild or moderate increases in afterload can produce a striking reduction in SV and subsequently precipitate an AHF episode.

**Fig. 3:**
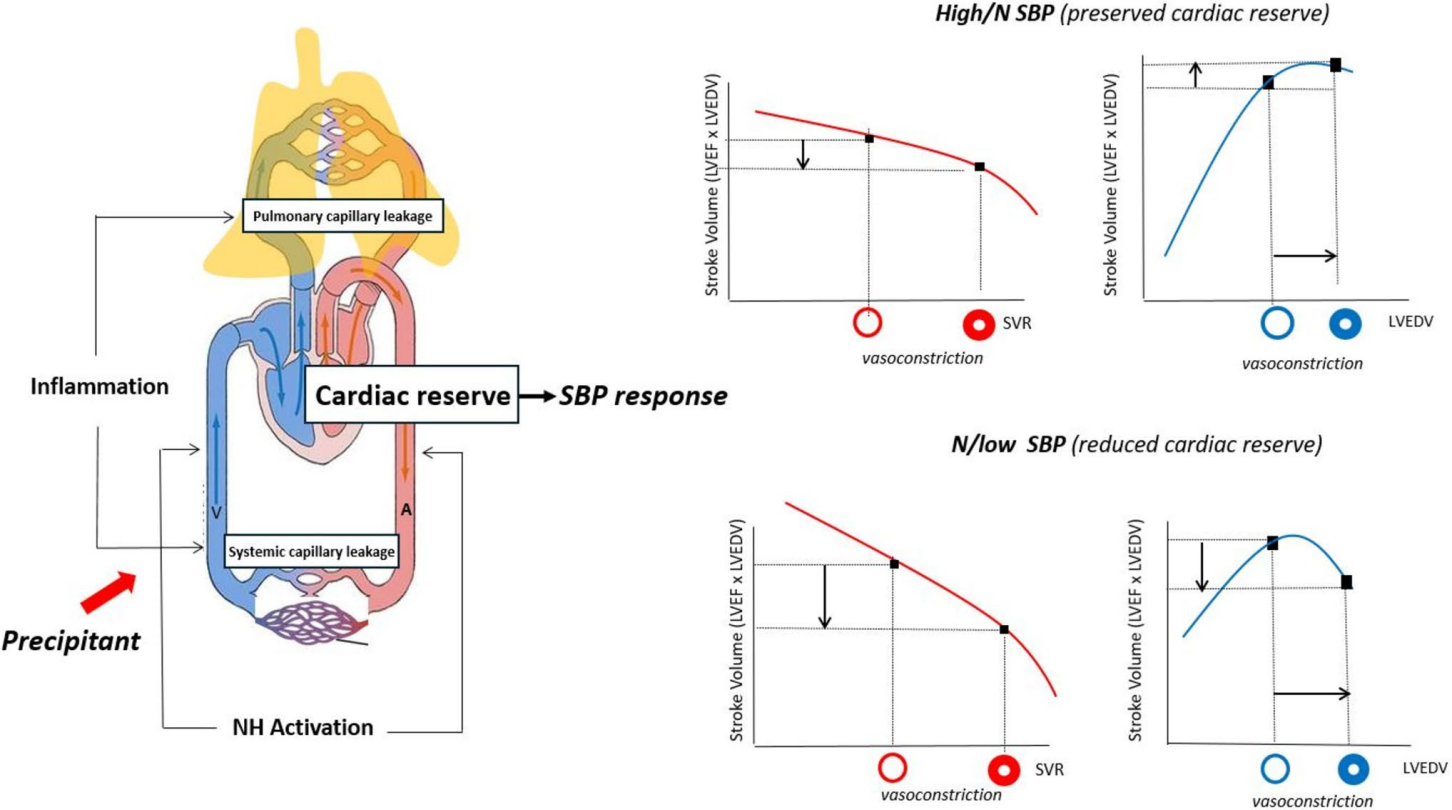
SBP response at presentation in AHF. Irrespective of the underlying etiology or type of precipitant, during AHF, enhanced neurohormonal activation favors vasoconstriction of both the arterial and venous compartments, which acutely challenges cardiac preload and afterload. This is combined with inflammatory-related leakiness of the vascular tree, most importantly the alveolar barrier, which leads to pulmonary congestion and peripheral oedema. In this milieu, the ability to increase SBP in the acute phase of HF regardless of etiology or LVEF may reflect a greater “cardiovascular reserve” that can be mediated by a greater ability to achieve a vasoconstrictive response and/or a greater myocardial contractile reserve, translating into greater cardiac output. These patients will benefit most from a vasodilator-based therapy without many diuretics because they mostly have fluid redistribution due to leakiness of blood vessels and afterload mismatch that shifts fluids to the pulmonary circulation. On the other hand, patients with low cardiac reserve usually react with low or normal SBP. Abbreviations: *AHF* acute heart failure; *HTN* hypertension; *LV *left ventricle; *LVEDV* left ventricular end-diastolic volume; *LVEF *left ventricular ejection fraction; *NH* neurohormonal activation; *SBP *systolic blood pressure; *SVR *systemic vascular resistance

In addition to these considerations based on hypertensive pathophysiology, high SBP may be a reactive stress response to various precipitants. Despite having high SBP at presentation, some AHF patients may not have a history of HTN or hypertensive pathophysiology. Irrespective of the underlying aetiology, any precipitant may amplify neurohormonal and inflammatory activation, leading to venous and arterial vasoconstriction, tachycardia, and increased vascular permeability. Depending on the baseline cardiac reserve and the magnitude and type of the precipitant, the CV response will be with low, normal, or high SBP (Fig. 3). In patients with non-recruitable or exhausted cardiac reserve, the response will be characterized by low or normal SBP, whereas in patients with potentially preserved cardiac reserve, the response will be associated with high SBP [36].

These pathophysiologic mechanisms have important therapeutic implications and should be considered when assessing patients with AHF and elevated BP [37].

High SBP may be the unique causal factor of decompensation, more commonly in HFpEF patients, often presenting as de novo AHF. Rapid improvement in clinical signs and symptoms by BP-lowering treatment suggests that high SBP is the main contributor to the pathogenesis of AHF. On the other hand, when high SBP is a reactive stress response to another precipitant (i.e., worsening ischemia, rapid rate AF, or infection), clinical status will improve only after addressing these conditions.

### Left ventricular ejection fraction

In patients with normal pre-existing cardiac function, a sudden increase in afterload leads to an increase in LV end-diastolic volume (LVEDV), activating the Frank-Starling mechanism and acutely compensating for the reduction in SV. There is a curvilinear relationship between LV end-diastolic pressure (LVEDP) and LVEDV, such that at high intracavitary pressures (LVEDP > 20 mmHg), the relationship between these two parameters reaches an extreme degree of steepness where further changes in LVEDP result in minimal LVEDV modifications [38, 39].

In states of reduced compliance, as seen in HF with preserved EF (HFpEF), ventriculo-arterial stiffness leads to heightened sensitivity to loading conditions, manifesting as an exaggerated rise in pressure in response to smaller volume increases and an inability to accommodate even normal volumes with acute increases in afterload [40, 41]. Such exhaustion of the preload reserve requires very high LV filling pressures (more than 30 mmHg), rendering sharp drops in SV with any further increase in aortic pressure [42].

Conversely, in patients with severely depressed LVEF, preload reserve is expected to be near maximally utilized even under resting conditions, with the LV operating on a depressed force–velocity curve. Thus, an afterload mismatch exists at baseline regardless of the aortic pressure. In this scenario, only a mild or moderate increase in afterload can produce a striking reduction in SV and may precipitate AHF. Therefore, in patients with “cardiac failure” and chronic volume overload, as opposed to “vascular failure,” an even smaller increase in arterial vascular tone and a correspondingly smaller rise in BP may trigger a significant increase in afterload leading to HF decompensation.

### Ventriculo-arterial stiffening

The aorta and proximal large vessels store about 50% of the LV stroke volume during systole, with further diastolic forwarding into the peripheral circulation. This dynamic process has a crucial role in the normal functioning of the LV as a hemodynamic pump and in the normal functioning of the resistance vessels [43].

As aortic distensibility declines, the resulting augmentation of forward pulsatile load leads to a subsequent rise in LVEDP, thereby compromising LV filling. In a non-compliant ventricle, this combined ventriculo-arterial stiffening amplifies pressure-load dependence, leading to disproportionate rises in LVEDP and cardiac workload in response to minor changes in blood volume [38, 40, 41].

Interestingly, this level of pressure-load dependence is not seen in patients with LV systolic dysfunction, further supporting the theory that the increase in both arterial elastance and end-systolic elastance, rather than intrinsic LV diastolic dysfunction alone, is what augments systolic pressure sensitivity to cardiac loading and exacerbates pressure responses during physical exertion [6, 40, 41].

### Myocardial ischemia

Patients with AHF and high SBP, with or without coronary artery disease (CAD), commonly have myocardial injury, which may be related to multiple factors, including abnormal flow dynamics, subendocardial ischemia from microvascular dysfunction, and coronary vasoconstriction [44]. Regardless of the mechanism, acute ischemia can alter both systolic and diastolic properties.

Although it was thought that the primary mechanism of AHF in patients with APO and high SBP was due to transient ischemic LV systolic dysfunction, Ghandi et al. showed no difference in LVEF or wall motion abnormalities before and after the resolution of the acute event in patients presenting with marked HTN and APO [31]. Thus, a worsening of the diastolic dysfunction, rather than transient systolic dysfunction, seems to be the mechanism responsible for the clinical picture in these patients [45]. Nevertheless, these findings do not refute the role of associated myocardial injury as a determinant or aggravating factor, given that most of these patients have HFpEF, which is commonly associated with impaired coronary flow reserve and reduced coronary microvascular density. When an acute rise in LVEDP occurs, these structural and functional alterations can compromise myocardial blood flow, thereby leading to subendocardial ischemia and myocardial injury [6]. In these patients, the degree of myocyte injury is associated with increased LV filling pressures, cardiac reserve depreciation, and an inability to adequately increase CO and oxygen consumption. These data are supported by a surprisingly high rate of positive stress tests among HFpEF patients with no angiographically proven significant epicardial coronary disease, highlighting the impact of increased LV mechanical wall stress on the coronary microcirculation [46]. In patients with known CAD, acute ischemia can trigger AHF decompensation via multiple factors, including neurohumoral activation, increases in LVEDP, reactive stress, an increase in SBP, and further deterioration of myocardial blood flow and cardiac function [47].

### Microcirculatory dysfunction

HTN induces three primary structural changes in the systemic microcirculation: vascular remodelling, structural modifications of resistance to small arteries and arterioles with a media-to-lumen ratio, and overproduction of type I collagen in the extracellular matrix [48]. These changes increase arterial stiffness and fibrosis and reduce vessel density. HTN activates mechano-sensing molecular mechanisms in endothelial and vascular smooth muscle cells (VSMCs), amplifying vascular remodelling [48]. The progressive fibrosis of the microvasculature reduces its capacity to respond to physiological changes, thereby exacerbating HTN and contributing to end-organ damage in systems such as the heart, kidneys, and brain [49]. LVH in HTN causes microvascular rarefaction, which leads to interstitial fibrosis and myocyte hypertrophy. This increases the intercapillary distance and reduces myocyte oxygen delivery [48]. Numerous experimental and clinical studies have reported that microcirculation is altered in patients with AHF, and the extent of microvascular abnormalities has been correlated with organ dysfunction and mortality in AHF [50].

### Inflammation

HTN and inflammation are physiologically connected. A low-grade immune response is essential for initiating and maintaining elevated BP. Immune cells that accumulate in the hypertensive kidney can influence the severity of HTN by altering the functions of cells in the renal vasculature and the nephron that modulate renal vascular resistance and sodium handling [51, 52]. IL-1β is an upstream inflammatory factor of IL-6 and CRP (C-reactive protein), with all three factors being closely involved in the progression of HTN [53]. Similarly, inflammation contributes to the pathogenesis and progression of AHF across the entire spectrum of LVEF [51–56]. Activation of systemic inflammation is even more evident in AHF, where elevated hs-CRP concentrations (12.6 mg/l) were observed in the ASCEND-HF trial and independently associated with poor prognosis [57]. Inflammation in AHF is associated with reduced NO availability, increased vascular permeability, microvascular rarefaction, and organ damage, subsequently enhancing the reactive stress response and contributing to the severity of congestion [58]. In the CORTAHF trial, which enrolled patients with AHF and high CRP, prednisone was associated with decreased CRP levels and improved congestion [59, 60].

### Vulnerability of pulmonary capillaries

The extracellular matrix, mainly the type IV collagen in the lamina densa of the basement membrane, is the main stress-bearing component of the blood-gas barrier. In a study conducted by Lee et al. in patients with chronic pulmonary oedema secondary to cardiac disease, the capillary basement membrane fragmentation was seen only in patients with pulmonary wedge capillary pressure (PWCP) greater than 35 mmHg for periods exceeding 6 years [61]. Even in such extreme circumstances, the structural alterations were only mild, proving a powerful defence mechanism against mechanical stress [61]. In contrast, the capillary endothelial and alveolar epithelial layers demonstrate significantly greater vulnerability, undergoing significant ultrastructural changes at significantly lower hydrostatic pressures [6, 62]. Traditionally, disruption of the blood-gas barrier equilibrium is reflected as one of two types of alveolar oedema: cardiac or hydrostatic pulmonary oedema, containing protein-poor fluid, and non-cardiac or high-permeability pulmonary oedema, containing protein-rich fluid. However, this binary classification may not be as appropriate as initially anticipated, as evidence suggests that various cardiovascular pathological states can lead to a mixed phenotype of both forms of oedema [62].

Initially, disruption of the Starling equilibrium drives fluid transudation from the capillary lumen into the interstitial alveolar wall and, in some cases, the alveolar spaces, resulting in hydrostatic pulmonary oedema. As capillary pressure continues to rise, damage to the pulmonary capillary wall occurs, resulting in increased permeability and more significant protein loss from the capillary. Finally, at very high pressures, stress-induced failure of the barrier occurs, resulting in the disruption of one or more of its layers, which ultimately leads to high-permeability alveolar oedema. Hence, in extreme cases, high-permeability pulmonary oedema can be of cardiac origin [62]. Another noteworthy consideration is the continuous remodelling of the alveolar-capillary membrane observed in patients with chronic cardio-centric pulmonary oedema. Several studies on such patients have demonstrated an increase in type II alveolar cells and irregular thickening of alveolar epithelial and capillary basement membranes. This increase in type II alveolar cells may enhance surfactant production, which helps stabilize alveolar spaces and promote alveolar fluid transport out of the alveoli through various ion channels [61]. These adaptations may serve as a protective response against sustained elevations in capillary pressure.

## HTN emergencies

In the recent ESC Guidelines, HTN emergency is defined as a BP of ≥ 180/110 mmHg associated with acute HTN-mediated organ damage (HMOD), often in the presence of symptoms [63]. In these situations, the rate of BP increase is more critical than the absolute BP value. Rapid shifts in fluid balance caused by RAAS activation and diastolic dysfunction ultimately lead to symptoms such as flash pulmonary oedema [64]. Therefore, it is essential to identify and address underlying conditions that can contribute to recurrent episodes of HTN. For example, renal artery stenosis (RAS) is often an underrecognized cause of recurrent hypertensive pulmonary oedema, otherwise known as Pickering syndrome. Compromised renal perfusion leading to abnormal RAAS activation in RAS leads to recurrent, life-threatening episodes of hypertensive pulmonary oedema. In such cases, revascularization through percutaneous artery stenting has demonstrated significant clinical benefits in patients with hemodynamically significant RAS.

It is important to note that all HTN emergencies are potentially life-threatening and require immediate and careful intervention to reduce BP, often with IV therapy. Acute HMOD includes stroke (ischaemic or haemorrhagic), acute HTN microangiopathy and encephalopathy, cardiogenic pulmonary oedema, coronary ischaemia, and acute aortic disease. Furthermore, these acute manifestations of organ damage from severe acute HTN may be associated with other clinical conditions that typically warrant urgent BP reduction, e.g., acute onset of aortic dissection, myocardial ischaemia, eclampsia, or HF [65].

The recent ESC Guidelines set a threshold BP of ≥ 180/110 mmHg to define hypertensive emergency [63].

## Therapeutical management

Acute management of AHF and high SBP is similar to the general management of patients with AHF, including non-invasive ventilation (NIV), vasodilators, and diuretics. Treatment decisions should be individualized based on a range of clinical factors, such as the degree of respiratory distress, predominance of either congestion or HTN, LV systolic and diastolic performance, history of HTN, baseline antihypertensive treatment, and associated comorbidities [10]. For example, in patients with isolated diastolic dysfunction or “vascular failure” presenting with extremely high BP levels, an immediate reduction of SBP under 140 mmHg is recommended [34, 43, 61].

In more critical scenarios, such as HTN emergencies, immediate IV therapy to reduce BP is required. In this clinical scenario, the 2017 ACC/AHA Guidelines and a recent ESC scientific statement recommend against rapid, uncontrolled, or excessive BP reduction, emphasizing a controlled approach to lowering BP safely while avoiding hypotension [37, 65]. SBP should be reduced by no more than 25% within the first hour; then to 160/100 mmHg within the next 2 to 6 h if stable; and gradually to normal levels over 24 to 48 h [65, 67]. Data from the REALITY-AHF trial showed that early administration of IV vasodilator therapy without causing excessive SBP reduction (SBP < 25%) was associated with improved diuretic response and reduced 1-year mortality [68]. However, initiating low-dose nitroglycerin (NTG) with gradual titration, as recommended by current guidelines, may take excessive time and delay the therapeutic effect. Different algorithms for high-dose NTG have been proposed in small studies, showing promising safety and efficacy; however, the optimal degree of BP reduction in the absence of HMOD remains uncertain [69–71].

Conversely, in patients with HT-AHF with systolic LV dysfunction, the primary precipitant factor for an episode of ADHF is very often volume overload secondary to hemodynamic pump failure with mild or moderate increments in afterload. Hence, therapy should primarily be directed at preload reduction. In these cases, diuretics are considered the first option for treatment, offering a better efficacy and safety profile, while vasodilators are regarded as more of an adjuvant therapy. When IV vasodilators are used, careful titration should be done to avoid hypotension [45].

### Non-invasive ventilation (NIV)

The role of positive-pressure ventilation in patients with respiratory distress has long been recognized, with the first reports dating back to 1938 [72]. Its efficiency resides in restoring functional residual capacity through alveolar recruitment, thereby mitigating right-to-left intrapulmonary shunt and improving oxygenation and lung mechanics. Positive intrathoracic pressure also decreases preload and LV afterload, both of which are beneficial in patients with intravascular volume overload.

NIV, especially continuous positive airway pressure, is part of standard medical therapy and is advantageous over standard medical treatment alone. Meta-analyses have demonstrated that it prevents metabolic abnormalities and reduces intubation rates in AHF patients, with neutral effects on mortality [73, 74]. The use of NIV is a valuable intervention in patients with severe respiratory distress (respiratory rate > 25 breaths/min, SpO2 < 90%) and, in most cases, should be regarded as first-line therapy [1].

### Vasodilators

Over the last decade, several RCTs have demonstrated no significant benefit of vasodilator use in improving outcomes in AHF, resulting in a downgraded recommendation for vasodilator use in the 2021 ESC Guidelines. However, they may be considered in patients with AHF and SBP > 110 mmHg as initial therapy to improve symptoms and reduce congestion (class IIb indication, level of evidence B) [1]. Though evidence of the safety and efficacy of vasodilator therapy in AHF is relatively scarce, data suggests that it has beneficial effects in some AHF phenotypes [70, 75–77].

The PRONTO trial, which enrolled 113 AHF patients with SBP > 160 mmHg, demonstrated that treatment with IV clevidipine significantly improved dyspnea compared to the standard of care using other IV vasodilators [78]. However, the effects on 30-day hospitalizations were non-significant [78]. GALACTIC compared usual care (including the use of nitrates) with early intensive and sustained oral vasodilation and found no beneficial effect from vasodilators [79]. The cluster-randomized ELISABETH trial investigated the use of nitrates in the emergency department in an AHF population. Only a quarter of patients in the usual care arm received IV nitrates compared to 96% in the intervention arm. There were no differences in the number of days alive and out of the hospital at 30 days [63]. A meta-analysis of 46 RCTs (28,374 AHF patients) also demonstrated a lack of impact of vasodilators on hard outcomes in AHF. However, their use was associated with a reduced risk of intratracheal intubation, despite considerable heterogeneity of the data [80].

**Fig. 4:**
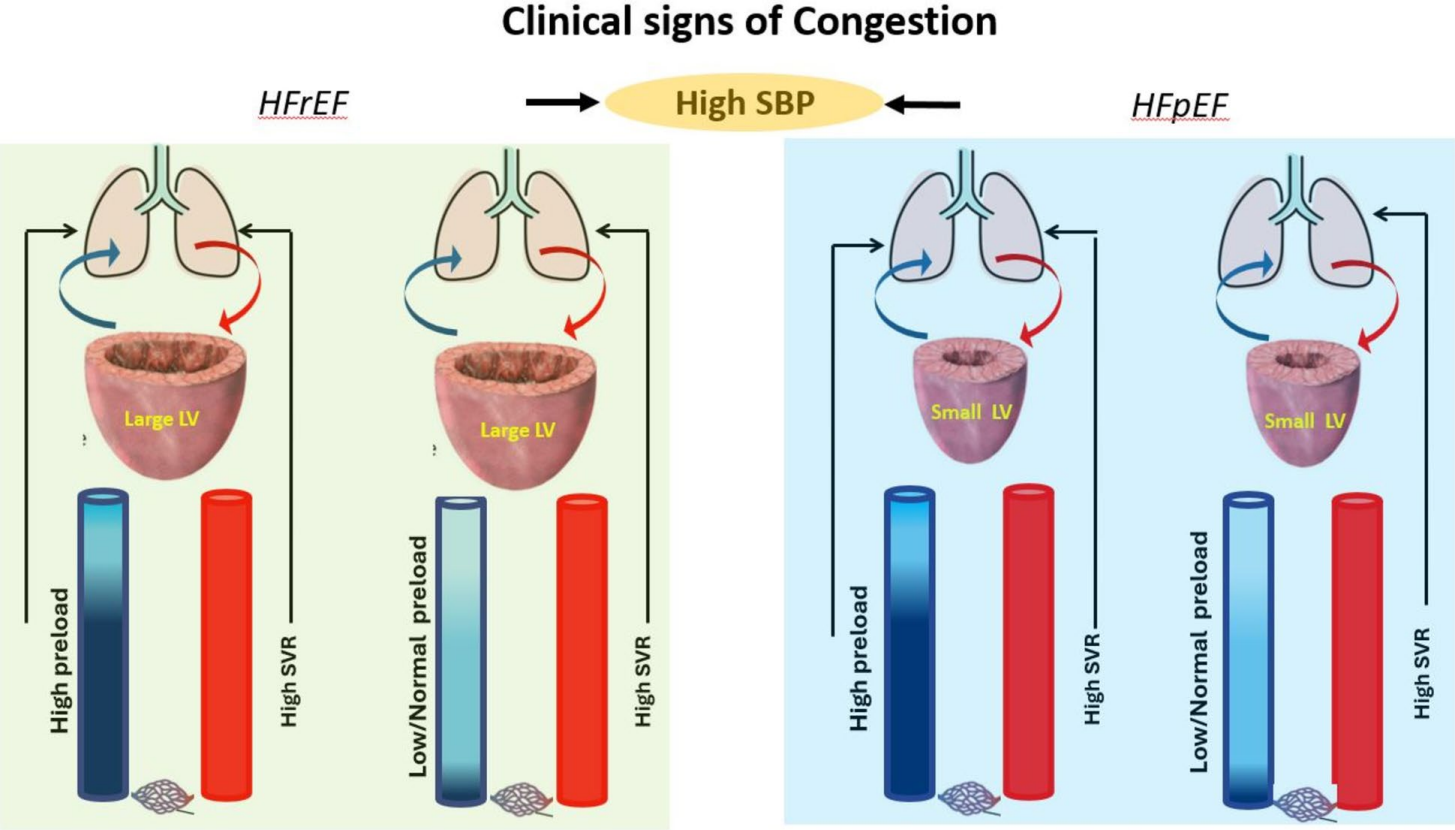
In AHF with high SBP at presentation, identifying different categories based on LVEF, preload, and afterload could personalize treatment. Patients with AHF and high SBP at admission may present with various combinations of alterations of preload and afterload. High preload with an increased LVEDV, due to increased venous return and reduced LV compliance, can be encountered in both HFpEF (increased LVEDP) and HFrEF patients (increased LVEDV). Drugs like diuretics and nitrates that reduce volume overload and venous return will lower preload. High SVR is likely present in AHF and very high BP, as subsequent vasoconstriction is the leading cause of high SVR. In these cases, management of high SVR decreases the LV workload, leading to high CO and better end-organ perfusion. Vasodilators such as ACE inhibitors, ARBs, calcium channel blockers, and high doses IVnitrates are the treatments of choice for reducing SVR. Use of IV diuretics in patients with high SVR and low preload is not advisable. In patients with both high preload and afterload, the treatment should be initiated with IV nitrates and then adjusted according to SBP response and evolution of congestion. Abbreviations: *ACE* angiotensin-converting enzyme; *AHF* acute heart failure; *ARBs* angiotensin II receptor blockers; *BP* blood pressure; *CO* cardiac output; *HFpEF* heart failure with preserved ejection fraction; *HFrEF* heart failure with reduced ejection fraction; *LV* left ventricle; *LVEF* LV ejection fraction; *LVEDP* LV end diastolic pressure; *LVEDV* LV end diastolic volume; *SVR* systemic vascular resistance

**Fig. 5:**
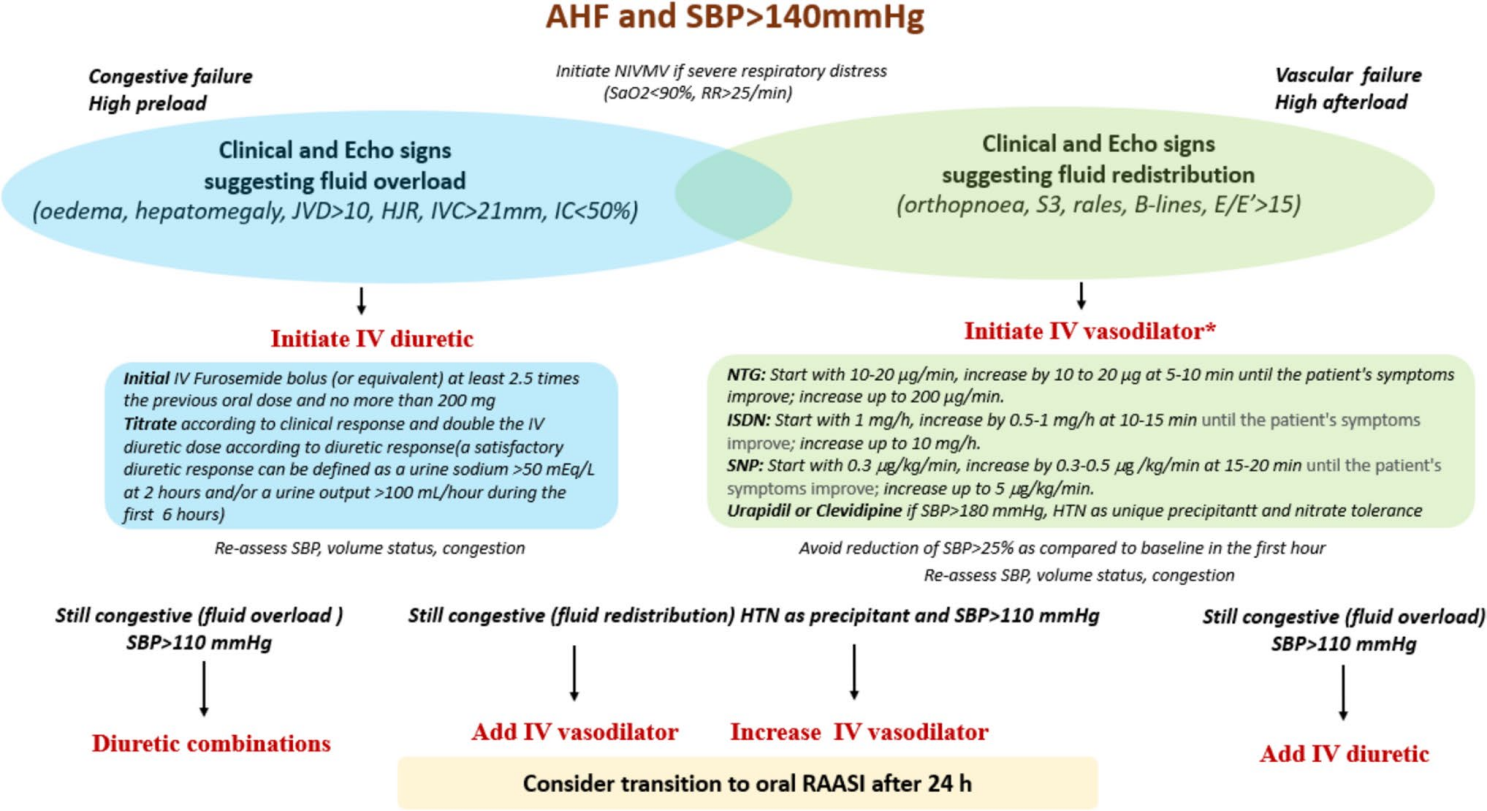
Practical approach in patients with AHF with SBP>140 mmHg. Abbreviations: *AHF* acute heart failure; *HJR* Hepatojugular Reflux; *IC* Inspiratory Capacity; *ISDN *Isosorbide Dinitrate; *IV* Intravenous; *mEq/L* milliequivalents per Liter; *IVC* Inferior Vena Cava; *JVD* Jugular Venous Distention; *NTG* Nitroglycerin; *RAASI* Renin-angiotensin-aldosterone-system inhibitors; *S3* Third Heart Sound; *SBP* Systolic Blood Pressure; *SNP* Sodium Nitroprusside

IV nitrates (nitroglycerine (NTG)/isosorbide dinitrate (ISDN)/sodium nitroprusside (SNP)) in patients with AHF and high SBP are known to improve symptoms and reduce congestion through a dual effect: decrease of venous tone with reduction of preload and decrease of arterial tone with subsequent afterload reduction. This was illustrated in the VMAC trial, where patients who received IV nesiritide or IV NTG were associated with significantly more reductions in pulmonary capillary wedge pressures and greater degrees of dyspnea relief than placebo [81]. Notably, nitrates produce a marked vasodilator effect on veins and a modest vasodilator effect on arteries because arterial dilation is only obtained at higher doses (≥ 150–250 µg/min) [82]. Thus, nitrates produce a marked vasodilator effect on veins and a modest vasodilator effect on arteries unless the dose is high. This distinction is clinically relevant when deciding whether to tailor initial treatment towards preload or afterload reduction, as essential pathophysiological differences exist between patients with high PCWP versus those with high SVR (Figs. 4 and 5).

In contrast, SNP combines arterial and venous dilator properties. It markedly reduces LVEDP and PCWP to a greater extent than NTG while minimally affecting the transpulmonary gradient. Subsequent afterload reduction due to arterial vasodilatation significantly increases SV and CO, while venous dilation reduces central venous pressure [83].

In patients with APO, high-dose ISDN was superior to IV diuretics in relieving pulmonary congestion symptoms and reducing the need for mechanical ventilation [84]. This advantage may be related to the rapid onset of ISDN, which peaks at 5 min after IV administration. In contrast, IV furosemide induces diuresis after 30 min, with its peak effect occurring 1–2 h after administration.

Interestingly, although nitrates are widely used in the ED in patients with AHF and high BP, the ELISABETH trial found no differences in short-term outcomes (number of days alive and out of the hospital at 30 days) with early nitrate boluses compared to standard-of-care [85].

*ACEi* are established therapies for chronic HF, but the evidence of their use in patients with hypertensive AHF is limited to a retrospective cohort study of AHF patients who received IV enalaprilat [86].

Dihydropyridine-type calcium-channel blockers (nifedipine, nicardipine, clevidipine) are potent agents for lowering BP. However, their use in AHF is poorly studied and is considered less optimal for AHF patients due to their negative inotropic effects and unpredictable rapid reductions of SBP. Among these agents, clevidipine has shown favorable hemodynamics. It is a rapidly acting, arterial-selective IV calcium-channel blocker with limited effects on capacitance vessels, and in patients presenting to the ED with AHF and SBP ≥ 160 mm Hg, it has been shown to reduce SBP and improve dyspnea more effectively than standard-of-care IV anti-hypertensive therapy [78, 87]. Althoughclevidipine has shown promising results in BP control, its availability is limited outside North America and Western Europe.

Nevertheless, comprehensive vasodilator strategies, as investigated in the GALACTIC trial, did not demonstrate significant improvements in composite outcomes (including all-cause mortality and rehospitalizations for AHF) compared to usual care over a 180-day follow-up period [79].

Alpha-receptor antagonists have also been studied in the management of AHF, particularly in the elderly. Intravenous administration of urapidil may be added, particularly in pregnancy and elderly patients. In a multicentric RCT, investigators found that urapidil, a dual alpha1-adrenergic receptor antagonist and 5-HT1A agonist, demonstrated superior efficacy and safety compared to NTG in elderly patients with HF complicated by HTN and diabetes [88]. Not only did urapidil provide a greater reduction in SBP, but it was also associated with more significant improvements in LVEF, cardiac index, NT-proBNP reduction, and LVEDP compared to nitroglycerine. However,urapidil is available in some European countries but not globally.

### Diuretics

Loop diuretics are the cornerstone of treatment in AHF patients with signs of fluid overload and congestion [1]. They are the first-line therapy for patients with AHF, elevated SBP, and LV systolic dysfunction with signs of volume overload, where rapid preload reduction is the primary goal. For an appropriate therapeutic response, the initial IV dose is recommended to be 2.5 times higher than the patient’s prior oral dose (Fig. 5) [66, 89].

As previously mentioned, ventriculo-arterial stiffening in patients with “vascular failure” leads to enhanced pressure-load dependence [41]. In such cases, the acute rise in LVEDP and cardiac workload can occur with minor changes in intracardiac volume. Therefore, the use of diuretics in these patients may be inappropriate and potentially harmful by causing further deterioration of the cardio-renal axis via neurohormonal activation [90]. In these patients, vasodilators such as high-dose ISDN may be more beneficial, as they have demonstrated superiority over diuretics in patients with AHF and APO [84]. In AHF patients with high SBP who present clinical signs of overall fluid overload, a combination of IV mixed vasodilator (e.g., IV NTG) and low-dose IV diuretics may be used as initial therapy (Fig. 5).

### Morphine

The primary mechanism driving morphine administration in AHF is believed to involve the reduction of sympathetic activity, the anxiolytic effects, and the consequent preload reduction [91].

However, in the largest sub-analysis of the ADHERE registry, AHF patients treated with morphine were more likely to receive inotropes and vasodilators, require mechanical ventilation (15.4% vs 2.8%), experience longer median hospitalization (5.6 vs 4.2 days), have more ICU admissions (38.7% vs 14.4%), and exhibit greater mortality (13.0% vs 2.4%) (all *p* < 0.001) compared to patients without morphine treatment. In fact, morphine use was identified as an independent predictor of mortality (*p* < 0.001) [92, 93]. Similarly, the MIMO (Midazolam versus Morphine) trial demonstrated a significantly higher rate of secondary adverse events in the morphine group when compared to intravenous midazolam [94]. Thus, other vasodilators are preferred over morphine in this population.

## Discussion

The 2021 ESC-HF Guidelines’ exclusion of HT-AHF as a distinct clinical profile marks a significant shift in classifying and managing AHF patients. This decision diverges from earlier guidelines and several registries that recognized HT-AHF as a distinct clinical entity, highlighting the evolving nature of HF understanding and treatment.

### Overlapping clinical profiles

One of the primary reasons for excluding HT-AHF from the latest guidelines is its considerable overlap with other AHF profiles, such as ADHF and APO. This overlap poses challenges in clinical diagnosis and management, as these conditions share many clinical features, such as elevated BP, dyspnoea, and signs of fluid overload. In registries, about 70% of patients with HT-AHF have a history of HF, leading to their classification as ADHF rather than HT-AHF. If the classification is mutually exclusive, patients presenting with high SBP and HTN pathophysiology and a history of HF may be classified as ADHF rather than HT-AHF. Similarly, a significant proportion of HT-AHF patients in the same registries present with signs and symptoms of fluid overload. These patients often present with severe dyspnoea, respiratory distress, and respiratory insufficiency, which are overlapping features of APO. Furthermore, while preserved LVEF and LVH are common in other clinical profiles, some patients with HT-AHF may also present with reduced LVEF.

Increasing the specificity and homogeneity of the HT-AHF definition requires the addition of more diagnostic criteria to minimize overlap with other clinical phenotypes. For instance, additional criteria such as the de novo occurrence of dyspnea and rales without respiratory distress, in the context of high SBP at presentation, will significantly reduce the sample size of the population in this category (Fig. 1). In the ESC-HF-LT registry [2], redefining HT-AHF to include the concomitant presence of SBP > 140 mmHg at presentation and high SBP as a precipitating factor would retain just 61% of those initially classified in this category. However, this refinement would not eliminate overlap with other clinical profiles, as 22% of ADHF and 18% of APO patients also meet these criteria. Conversely, further restricting the definition to patients with different stages of HTN pathophysiology risks excluding patients with other aetiologies, such as valvular heart disease, who may experience an AHF episode in the context of high SBP [95]. The current understanding of AHF clinical profiles suggests that it represents a continuum of clinical entities with different presentations, depending on the underlying aetiology and precipitating factors.

### Geographic variability

Geographic variability in the diagnosis and classification of HT-AHF has also contributed to its exclusion. Studies have shown that the prevalence and recognition of HT-AHF can vary significantly by region, influenced by differences in clinical practice, interpretation of symptoms, and thresholds for hospital admission [96].

### Pathophysiology

Irrespective of the clinical phenotype, alteration of ventricular-arterial coupling, microcirculatory dysfunction, low-grade inflammation, and sensibility of pulmonary capillary vessels contribute to increased sensitivity to triggers, severity, and persistence of symptoms in any AHF phenotype. Defining HT-AHF suggests a direct and unique causal relationship between acute elevations in afterload secondary to SBP surge and symptom occurrence, which can be difficult to demonstrate in heterogeneous conditions such as AHF.

### Complex aetiologies

HT-AHF may be associated with multiple aetiologies, challenging its definition as a standalone category. High SBP at presentation can result from various acute triggers, including myocardial ischemia, arrhythmias, or exacerbations of underlying hypertensive heart disease. This complexity suggests a more granular classification based on pathophysiology and precipitating factors, rather than just BP and symptoms, might be more clinically useful.

### Clinical and diagnostic challenges

Creating a separate category for HT-AHF may diminish the overall clarity of the classification system. The symptoms of HT-AHF, such as severe dyspnoea and fluid overload, are not unique and can lead to misclassification with other types of HF. Moreover, relying on SBP alone to define this group may oversimplify the diverse pathophysiological processes involved in AHF.

### Management implications

HT-AHF management does not differ substantially from other forms of AHF, where rapid BP control is necessary. However, defining it as a distinct entity may promote a treatment pathway emphasizing vasodilator therapy, for which there is limited evidence in the broader context of AHF without a clear HTN component. While vasodilators are crucial for managing HTN emergencies, its utility extends to a broader spectrum of AHF phenotypes—especially in improving hemodynamics in patients without volume overload.

### Prognostic considerations

Studies and registries indicate that patients categorized under HT-AHF tend to have better short-term outcomes than those with other AHF phenotypes. HT-AHF patients exhibit lower 1-year mortality at 6 months post-discharge compared to other clinical profiles [2]. This observation raises questions about the severity and risk profile of this condition, prompting a reconsideration of whether HT-AHF warrants its own distinct classification. However, these outcomes must be interpreted with caution. Many cited studies have not consistently adjusted for confounding variables such as age, renal function, or comorbidity burden. Only the ESC-HF-LTregistry [2] reported multivariable adjustment using Cox proportional hazard models, adjusting for clinically relevant confounders such as age, gender, CKD, chronic obstructive pulmonary disease, and cancer.

Although high SBP is the hallmark of HT-AHF, no clinical studies have demonstrated a significant association between high SBP at presentation and either short- or long-term outcomes. This distinction is particularly noteworthy as the converse relationship holds true: low SBP at presentation is associated with adverse outcomes. The lack of association between high SBP and significant outcomes contrasts with the prognostic associations observed in other AHF phenotypes. For example, the severity of hypoperfusion in CS, the severity of respiratory distress in APO, and the severity of RV dysfunction in isolated right HF have all been shown to correlate with patient outcomes [97].

## Limitations

The data summarized herein are predominantly derived from European registries and North American reviews or consensus documents. Regional variability in hypertension prevalence, emergency care protocols, and drug availability can significantly affect diagnosis and outcomes. These differences limit the global generalizability of our findings and underscore the need for regionally inclusive trials.

## Implications for future research and guidelines

Future clinical trials should more closely examine the complex interplay between HTN and HF, with an emphasis on exploring tailored interventions that not only address the underlying pathophysiology of the disease but also modulate the acute CV response to an increase in SBP. Also,clinicians should actively distinguish whetherHTN serves as the underlying primary aetiology, a comorbidity, or a precipitating factor, given its potential to influence therapeutic choices and prognostic expectations.

Moreover, the discrepancy between low in-hospital mortality and high 1-year mortality highlights the need for research focused on strategies to stabilize the cardiovascular substrate during and after the acute episode, thereby reducing vulnerability to subsequent precipitating events.

The current decision to exclude HT-AHF as a distinct phenotype underscores the need for continued research on how different manifestations of HF are best classified and managed. It emphasizes the need for a dynamic classification system that can adapt to new scientific insights and more accurately reflect the clinical realities of treating AHF. Unexplored research areasincludeinternational registries capturing regional variability and underrepresented areas, registries capturing BP trajectories and biomarker evolution during AHF, real-world studies of vasodilator strategies in de novo HF vs. ADHF, and prospective trials validating HT-AHF definition basedon EF categories (HFrEF or HFpEF) in the large groupof patients with AHF and high SBP. Also, future studies should focus on delineating the impact of high BP in AHF through more homogeneous populations where hypertensive pathophysiology predominates and progressive fluid overload is excluded, thereby isolating high SBP as a unique precipitant.

## Conclusions

The ongoing evolution of HF classifications reflects a shift towards a more phenotype-guided treatment approach. This involves recognizing the heterogeneity of the different AHF clinical profiles and tailoring interventions based on individual patient characteristics rather than rigidly adhering to a broad classification that may not fully capture the nuances of the disease. When high SBP is a component of AHF clinical presentation, IV vasodilators may be used to rapidly improve clinical signs and symptoms, facilitating a transition to clinical stability and allowing for the early implementation of life-prolonging therapies. Future research should aim to identify specific clinical contexts in which hypertensive status directly influences long-term outcomes in AHF.

## Data Availability

No datasets were generated or analysed during the current study.
