# Correction to: Hypertensive acute heart failure: a critical perspective on definition, epidemiology, pathophysiology, and prognosis—a narrative review: a joint session with the Romanian Society of Cardiology (part II)

**DOI:** 10.1007/s10741-025-10570-7

**Published:** 2025-11-07

**Authors:** Oliviana Geavlete, Sean P. Collins, Alexandre Mebazaa, Linda Ye, Alberto Palazzuoli, Laura Antohi, Jan Biegus, Matteo Pagnesi, Petar Seferovic, Razvan I. Radu, Avishay Grupper, Oscar Miro, Beth Davison, Magdy Abdelhamid, Marija Polovina, Mitja Lainscak, Marianna Adamo, Gad Cotter, Gianluigi Savarese, Mehmet Birhan Yilmaz, Maurizio Volterani, Giuseppe M. C. Rosano, Javed Butler, Andrew P. Ambrosy, Ovidiu Chioncel

**Affiliations:** 1 Emergency Institute for Cardiovascular Diseases ‘Prof. C.C. Iliescu’, 258 Fundeni Street, 2 District, 022328 Bucharest, Romania; 2 University of Medicine Carol Davila, Bucharest, Romania; 3 Department of Emergency Medicine, Education and Clinical Center (GRECC), Vanderbilt University Medical Center and Veterans Affairs Tennessee Valley Healthcare System, Geriatric Research, Nashville, TN USA; 4 Department of Anesthesiology and Critical Care and Burn Unit, Saint‑Louis and Lariboisiere Hospitals, FHU PROMICE, DMU Parabol, APHP Nord, MASCOT, Universite Paris Cite, Paris, France; 5 Department of Medicine, Kaiser Permanente San Francisco Medical Center, San Francisco, CA USA; 6 Cardiovascular Diseases Unit CardioThoracic and Vascular Department, Le Scotte Hospital Siena, University of Siena, Siena, Italy; 7 Department of Cardiology, Clinical Department of Intensive Cardiac Care, Faculty of Medicine, Institute of Heart Diseases, Wroclaw Medical University, Wroclaw, Poland; 8 Institute of Cardiology, ASST Spedali Civili, Department of Medical and Surgical Specialties, Radiological Sciences, and Public Health, University of Brescia, Brescia, Italy; 9 Serbian Academy of Sciences and Arts; Medical Faculty, University of Belgrade, Belgrade, Serbia; 10 Department of Cardiology, University Clinical Centre of Serbia, Belgrade, Serbia; 11 Faculty of Medical and Health Sciences, Department of Cardiovascular Medicine, Shamir Medical Center, Tel-Aviv University, Be’er Ya’akov, Israel; 12 Emergency Department, Hospital Clinic, IDIBAPS, University of Barcelona, Barcelona, Catalonia Spain; 13 Momentum Research Inc, Durham, NC USA; 14 INSERM UMR‑S 942 (MASCOT), Universite Paris Cite, Paris, France; 15 Heart Initiative, Durham, NC USA; 16 Faculty of Medicine, Kasr Al Ainy, Department of Cardiovascular Medicine, Cairo University, Giza, Egypt; 17 Faculty of Medicine, University of Belgrade, Belgrade, Serbia; 18 Division of Cardiology, General Hospital Murska Sobota, Rakican, Slovenia; 19 Faculty of Medicine, University of Ljubljana, Ljubljana, Slovenia; 20 Department of Clinical Science and Education, Sodersjukhuset, Karolinska Institutet, Stockholm, Sweden; 21 Faculty of Medicine, Department of Cardiology, Dokuz Eylul University, Izmir, Turkey; 22 Cardiopulmonary Department, IRCCS San Raffaele Roma, 00166 Rome, Italy; 23 San Raffaele Open University of Rome, Rome, Italy; 24 IRCCS San Raffaele Roma, Rome, Italy; 25 Baylor Scott and White Research Institute, Dallas, TX and University of Mississippi, Jackson, MS USA; 26 Department of Cardiology, Kaiser Permanente San Francisco Medical Center, San Francisco, CA USA; 27 Division of Research, Kaiser Permanente Northern California, 4480 Hacienda Drive, Pleasanton, CA 94588 USA

**Correction to: Heart Failure Reviews**

10.1007/s10741-025-10551-w

The author's family name was incorrectly given as Lainshack. The correct family name is Lainscak.

Furthermore, the affiliation 18 should be Rakican, not Rakiˇcan.

Also, Funding information for Prof. Giuseppe M.C. Rosano was added: "This study was supported by the Italian Ministry of Health (Ricerca Corrente) 20/1819 for Prof. Giuseppe MC Rosano". 

Lastly, the affiliations of the author “Giuseppe M. C. Rosano “ were incorrectly assigned. It should be: San Raffaele Open University of Rome, Rome, Italy and IRCCS San Raffaele Roma, Rome, Italy.

We apologize for these errors.

The original article has been corrected.

